# Effects of a kappa opioid receptor antagonist on delayed postoperative pain recovery in a novel mouse sleep disorder model

**DOI:** 10.3389/fpain.2025.1516935

**Published:** 2025-07-04

**Authors:** Hisakatsu Ito, Masaaki Kawakami, Masashi Yoshida, Sadamu Sugimoto, Tomonori Takazawa

**Affiliations:** ^1^ Department of Anesthesiology, Toyama University Hospital, Toyama, Toyama, Japan; ^2^ Department of Anesthesiology and Intensive Care Unit, Gunma University Hospital, Maebashi, Gunma, Japan

**Keywords:** postoperative pain, pain recovery, sleep disorder, kappa opioid receptor, norbinaltorphimine, dynorphin

## Abstract

**Introduction:**

Sleep disturbances have been shown to exacerbate pain sensitivity and prolong recovery from pain. However, conventional animal models of sleep disturbance, which involve physical disruptions, such as water or forced movement, might not fully represent modern human sleep disorders.

**Methods:**

We utilized a novel sleep disorder model, the perpetual avoidance of water on a wheel (PAWW) model, which induces spontaneous activity, chronic stress, and abnormal sleep–wake cycles in mice. We investigated the effects of a kappa opioid receptor (KOR) antagonist, nor-binaltorphimine (nor-BNI), on pain recovery in a postoperative pain model in mice in a state of disordered sleep. After 1 week of acclimation, the mice were housed in PAWW or regular cages for 2 weeks. Sleep conditions were evaluated using electroencephalogram and electromyogram recordings, and postoperative pain recovery following plantar incision was assessed using von Frey tests. We also examined the effects of nor-BNI on pain recovery.

**Results:**

The evaluation showed that PAWW housing significantly increased activity during the light phase, disrupted sleep patterns, and delayed postoperative pain recovery. The administration of nor-BNI alleviated the delayed pain recovery.

**Discussion:**

These findings suggest that sleep disorders, such as those modeled by PAWW, could delay postoperative pain recovery, and that KOR antagonists might provide therapeutic benefits in the management of delayed recovery of postoperative pain induced by sleep disorders.

## 1 Introduction

Clinical studies have reported that sleep disorders in perioperative patients exacerbate postoperative pain (1–3). However, well-evidenced treatments for this issue have not been established. While basic research is essential for developing novel treatments grounded in scientific mechanisms, traditional animal models of sleep disturbance used in such studies often fail to replicate the sleep disorders experienced by humans in modern society. The conventional animal models rely on physical stimuli or acute strong stress, such as single platform-on-water, forced movement, mechanical stimulation, and fear-conditioning, to interrupt sleep (4–6), which does not accurately mimic the more subtle sleep disorders associated with modern stressors.

To address these limitations, we utilized the perpetual avoidance of water on a wheel (PAWW) model. In this model, mice are housed in a running wheel set above shallow water, promoting chronic voluntary movement and disrupting their sleep–wake cycle without acute physical stress (7). Although the PAWW model induces chronic stress, its primary feature is disruption of the normal sleep–wake cycle via increased spontaneous activity. In this study, we focused on the sleep impairment aspect of the PAWW model as it pertains to postoperative pain recovery. While we acknowledge that stress and sleep disturbances are closely interrelated, our experimental design aimed to investigate the effects of stress-induced sleep disruption on pain recovery, rather than measuring stress responses.

Pain and sleep are interlinked through common brain regions such as the frontal cortex, hypothalamus, and brainstem (8, 9), and via shared neurotransmitters, including opioids (10–13), monoamines (14–17), and orexin (18, 19). Dysregulation of these pathways contributes to both sleep disturbances and pain sensitization. Accordingly, pharmacologic targets such as antidepressants (20, 21), orexin receptor antagonists (22), kappa opioid receptor (KOR) antagonists (8, 9), and melatonin receptor agonists (23) are promising candidates for addressing both conditions.

Recent evidence has highlighted the role of dynorphin/KOR signaling in the anterior cingulate cortex and hypothalamus is implicated in sleep fragmentation and emotional dysregulation. Systemic KOR antagonism restores normal sleep in chronic pain models without altering baseline sleep in sham animals (8, 9). Moreover KOR signaling has also been reported to induce mechanical hypersensitivity in rodent pain models (24, 25). Although the underlying mechanisms of these effects remain unclear, they might involve interactions between the anterior cingulate cortex, hypothalamic orexin neurons, and ascending monoaminergic systems in the brain stem (14, 15, 26).

In the present study,we investigated the effect of nor-binaltorphimine (nor-BNI), a selective and long-acting KOR antagonist, on postoperative pain recovery in mice experiencing sleep disturbance induced by PAWW housing.

## 2 Materials and methods

### 2.1 Animals

This study was carried out according to the principles of the Act on Welfare and Management of Animals and the Guidelines for Proper Conduct of Animal Experiments of the Science Council of Japan, and followed the Guiding Principles for the Care and Use of Laboratory Animals at the University of Toyama. The experiments involved 8-week-old male C57BL/6J mice (Japan SLC), maintained at 22–26°C with a 12 h light–dark cycle. Zeitgeber time (ZT) 0 and ZT 12 represent the light onset (07:00) and offset times (19:00), respectively. Food and water were provided *ad libitum*. Every effort was made to minimize the number and suffering of the animals used in the experiments.

### 2.2 Study design

Animals were randomly assigned to either the PAWW or control group before the start of the experiments, with allocation being independent of the baseline number of wheel rotations. There was no significant difference in baseline wheel rotations between the PAWW and control groups, confirming the equivalence of locomotor activity prior to experimental manipulation.

**Figure 1:**
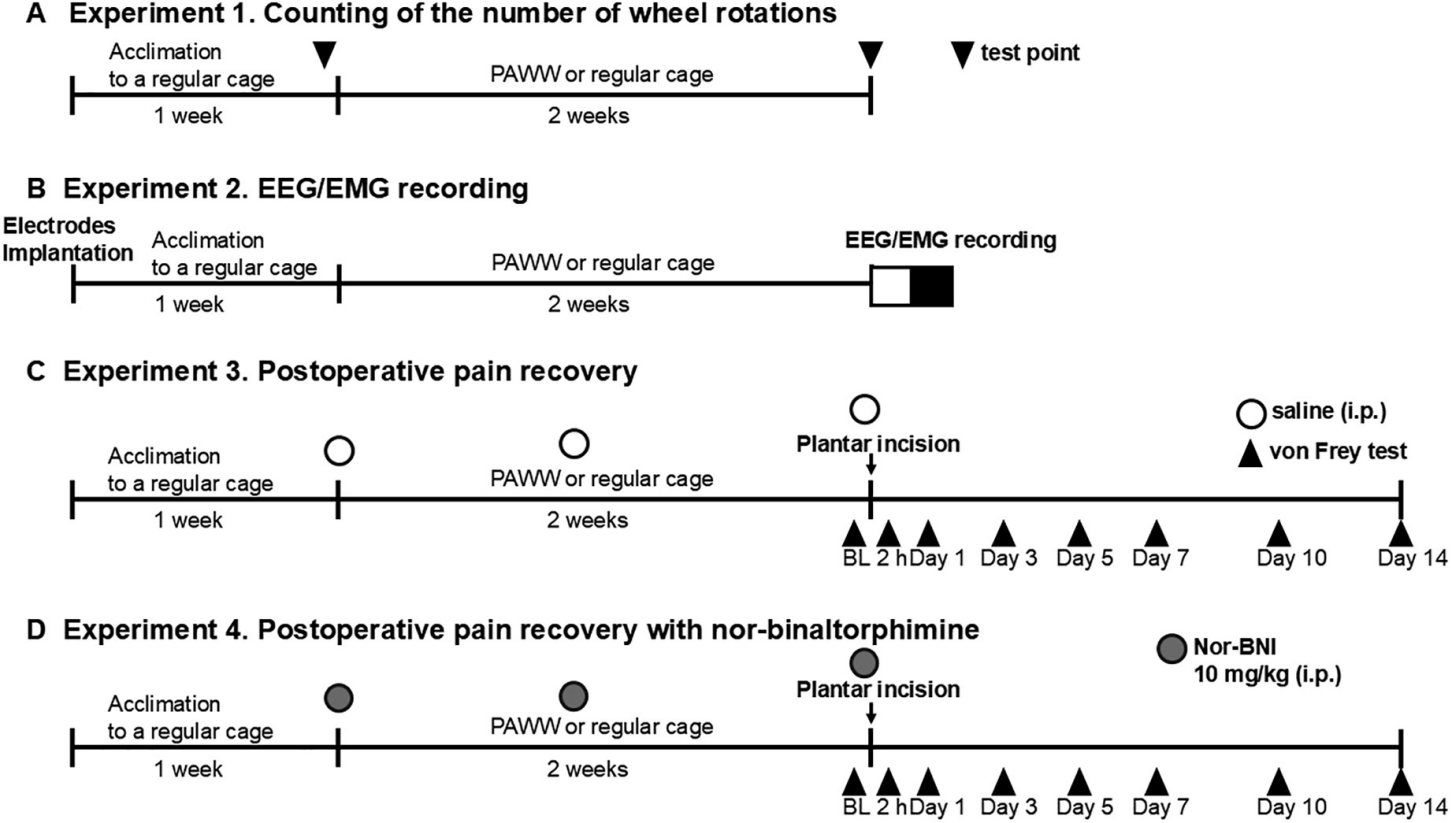
Experimental timeline. The timeline of each experiment, including the duration of housing in PAWW or regular cages and the timing of each treatment and measurement, is presented. The number of wheel rotations was counted after 1 week of acclimation in regular cages (baseline) and 2 weeks of housing in either PAWW cages or regular cages in experiment 1 **(A)** EEGs/EMGs were recorded after 1 week of acclimation in regular cages and 2 weeks of housing in either PAWW cages or regular cages in experiment 2 **(B)** Mice were subjected to von Frey tests at certain test points before and after the plantar incision surgery in experiment 3 **(C)** In experiment 4 **(D)**, they were treated with nor-BNI in addition to the same protocol as in experiment 3. BL, baseline; nor-BNI, nor-binaltorphimine; PAWW, perpetual avoidance of water on a wheel; EEG, electroencephalogram; EMG, electromyogram.

Experiment 1. After a 1-week acclimation period in regular wheel cages, the number of wheel rotations was counted for 24 h with an automatic counter. Mice were then either placed in PAWW cages or kept in regular cages for 2 weeks, based on their group allocation. Two weeks after the start of PAWW housing, the number of wheel rotations was recorded again for 24 h (Figure 1A).

Experiment 2. Mice underwent surgical EEG and EMG electrode implantation and were then placed in the regular wheel cages for the 1-week acclimation period. After acclimation, they were placed in PAWW cages or retained in the regular cages for 2 weeks. Thereafter, electroencephalograms (EEG) and electromyograms (EMG) were recorded for 24 h, as described below (Figure 1B).

Experiment 3. A plantar incision was performed as described below, after the mice were kept in PAWW cages or regular cages on the same schedule as in the previous two experiments. Von Frey testing was performed 2 h before surgery, and on days 1, 3, 5, 7, 10, and 14 post-surgery, to evaluate the course of postoperative pain recovery. Additionally, saline, which was also used as the vehicle for nor-BNI, was administered intraperitoneally before and one week after the start of PAWW rearing, as well as before baseline von Frey testing prior to the plantar incision surgery (a total of three doses), to maintain uniformity in experimental conditions and handling as in the next experiment, allowing for comparisons (Figure 1C).

Experiment 4. Mice breeding, plantar incision surgery, and von Frey tests were performed at the same schedule as in Experiment 3 in mice kept in both PAWW cages and regular cages. Additionally, 10 mg/kg of the long-acting kappa opioid receptor antagonist, nor-BNI (27), was administered intraperitoneally before and 1 week after the start of PAWW rearing, and before baseline von Frey testing prior to the plantar incision surgery (a total of three doses) to all the animals (Figure 1D).

### 2.3 Sleep disorder model

All the mice were individually housed in regular cages (length 225 mm × width 335 mm × height 210 mm), which are standard plastic cages equipped with a running wheel (diameter 140 mm × width 60 mm) and wood chip bedding, allowing them to sleep normally (RWC-15; Melquest, Toyama, Japan) for one week before exposure to stress. On the first day of exposure to stress, mice that were randomly allocated to the PAWW group were placed in the PAWW cage (SW-15-SD; Melquest, Toyama, Japan). The PAWW cage is a plastic cage (length 160 mm × width 180 mm × height 230 mm) that contains a centrally positioned, freely rotating running wheel (diameter 140 mm × width 60 mm), but with the mice being confined within the wheel. A water bottle and food dispenser were positioned within easy reach of the mice. The bottom of the PAWW cage is filled with water to a shallow depth, and no bedding is provided. However, the lower part of the wheel does not submerge in the water. Although the mice do not come in touch with the water, they tend to avoid the lowest position of the wheel. This aversion promotes sustained voluntary movement, leading to chronic sleep–wake cycle disturbances when housed in the PAWW cage for several weeks (7). The mouse group that continued to be kept in the regular wheel cage (RWC-15) without being transferred to a PAWW cage was designated as the control group. The cages were placed under conventional light on ventilated shelves, and the water in the PAWW cages was changed every day to keep the water clean. The number of wheel rotations of each mouse was recorded for 24 h with an automatic counter, then averaged for each 12 h light–dark phase.

### 2.4 EEG/EMG recording and sleep analysis

We recorded EEGs and EMGs to evaluate the sleep condition for 24 h, as previously described (12, 13). Briefly, mice were mounted in a stereotaxic head holder, and EEG and EMG electrodes were implanted for polysomnographic recordings (Pinnacle Technology, USA) under 3% isoflurane anesthesia before acclimation to the regular cage. Two stainless steel EEG recording screws were positioned 1 mm anterior to the bregma or lambda, both 1.5 mm lateral to the midline. EMG activity was monitored using Teflon-coated steel wires placed bilaterally into both trapezius muscles. The electrodes and the surgical site were covered with acrylic resin. The collected data were analyzed using appropriate software (SLEEPSIGN Kissei Comtec, Japan). Vigilance in every 10 s epoch was automatically classified into three stages, arousal, rapid eye movement (REM), and non-REM sleep; according to the standard criteria: (1) arousal was defined by a high EMG amplitude and low EEG amplitude; (2) REM sleep was defined by a low EMG amplitude, low EEG amplitude, and high *θ* wave activity; and (3) non-REM sleep was defined by a low EMG amplitude, high EEG amplitude, and high *δ* wave activity (16, 17). The defined sleep–wake stages were visually examined, and corrected if necessary.

### 2.5 Postoperative pain model

As a postoperative pain model, a 0.5 cm longitudinal incision was made through the mouse's plantar skin, fascia, and muscle under general anesthesia with 3% isoflurane (28). The incision site was sutured using 4-0 silk.

### 2.6 Mechanical allodynia assessment

Mechanical allodynia was assessed using von Frey filaments at different points before and after the plantar incision surgery. Mice were placed in chambers with wire mesh floors and allowed to habituate for 30 min. Von Frey filaments (range: 0.04–4.0 g) were alternately applied to the ipsilateral and contralateral hind paws until the filament buckled. The paw withdrawal threshold was determined using the up-down method, as previously described (29). Data were analyzed using UpDown Reader software (30). After completing the assessment, the mice were euthanized in accordance with institutional ethical guidelines depending on the requirements of the specific experimental protocol.

### 2.7 Preparation of nor-BNI

Nor-BNI (Abcam, CAS Number: 113158-34-2) was dissolved in a saline vehicle to a final concentration of 10 mg/ml. The solution was prepared before each experiment and stored at 4°C to maintain stability.

### 2.8 Statistical analysis

All data are expressed as the mean ± standard error of the mean (SEM). One-way ANOVA was used to analyze the number of wheel rotations. Repeated-measures two-way ANOVA was employed to analyze the percentage of sleep and the percentage of REM sleep, as these variables were measured in the same animals at multiple time points. Similarly, repeated-measures two-way ANOVA was applied to the von Frey test results. For all analyses, *post hoc* comparisons were performed using the Bonferroni multiple comparisons test. Statistical significance was set at *P* < 0.05. All data were tested for normality using the Kolmogorov–Smirnov test, with *P* < 0.05 considered indicative of deviation from a normal distribution. These statistical analyses were performed using Prism version 9.2 (GraphPad Software, La Jolla, CA, USA). Effect sizes were calculated from preliminary experiments, and the required sample size was estimated using G*Power 3.1 to achieve an actual power of >0.8. The effect sizes for comparisons in each experiment were estimated as follows: 1.8 for wheel rotations, 3.3 for sleep quantity determined by EEG, and 1.9 for the von Frey test in the postoperative pain model.

## 3 Results

The results of the various experimental conditions adopted to assess the effects of sleep deprivation, pain and nor-BNI treatment on postoperative pain recovery are shown below. All data met the assumption of normality (*P* > 0.05), as assessed by the Kolmogorov–Smirnov test.

**Figure 2:**
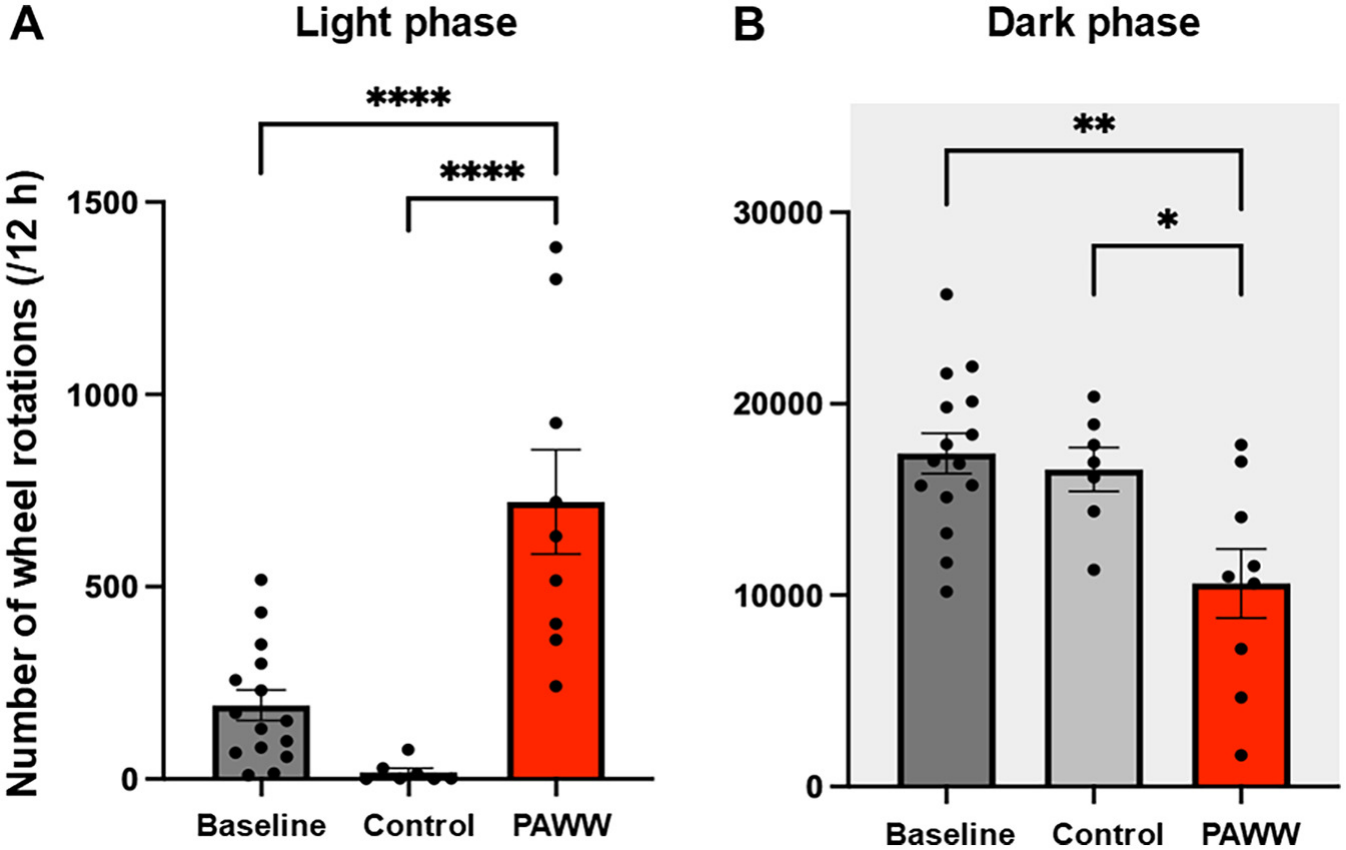
The number of wheel rotations of the mice in the PAWW and regular cages. **(A)** The number of wheel rotations recorded during the light phase (7:00–19:00). **(B)** The number of wheel rotations recorded during the dark phase (19:00–7:00). The measurements were taken during a 1-week acclimation period in regular cages (baseline, *n* = 15) and a 2-week housing period in either PAWW cages (*n* = 8) or regular cages (*n* = 7), and their average values were calculated. The results are presented as bar graphs. All data are expressed as the mean ± standard error of the mean. One-way ANOVA was used for statistical tests, and Bonferroni's multiple comparison test was performed as a post hoc test. Values were considered statistically significant at *P < 0.05, **P < 0.01, and ****P < 0.0001. PAWW, perpetual avoidance of water on a wheel.

In experiment 1 (Figure 1A), during the light phase, one-way ANOVA revealed a significant group effect [F(2,28) = 19.60, *P* < 0.00001, *η*² = 0.58]. *post hoc* Bonferroni tests showed that the number of wheel rotations in the PAWW cage group (*n* = 9) was significantly higher (720 ± 135.7 rotations) than that in the regular cage group (*n* = 7, 11 ± 17.1 rotations; *P* < 0.001) (Figure 2A). Additionally, the PAWW cage group exhibited a significant increase in light phase rotations compared to baseline (192 ± 39.7 rotations; *P* < 0.001) (Figure 2A). During the dark phase, one-way ANOVA revealed a significant group effect [F(2,28) = 7.37, *P* < 0.01, *η*² = 0.35]. *post hoc* Bonferroni tests showed that the number of wheel rotations in the PAWW cage group (10,611 ± 1,801 rotations) was significantly lower compared to the regular cage group (16,564 ± 1,141 rotations; *P* < 0.05) (Figure 2B). Similarly, the number of dark phase rotations in the PAWW cage group also showed a significant decrease compared to baseline (17,403 ± 1,058 rotations; *P* < 0.01) (Figure 2B).

**Figure 3:**
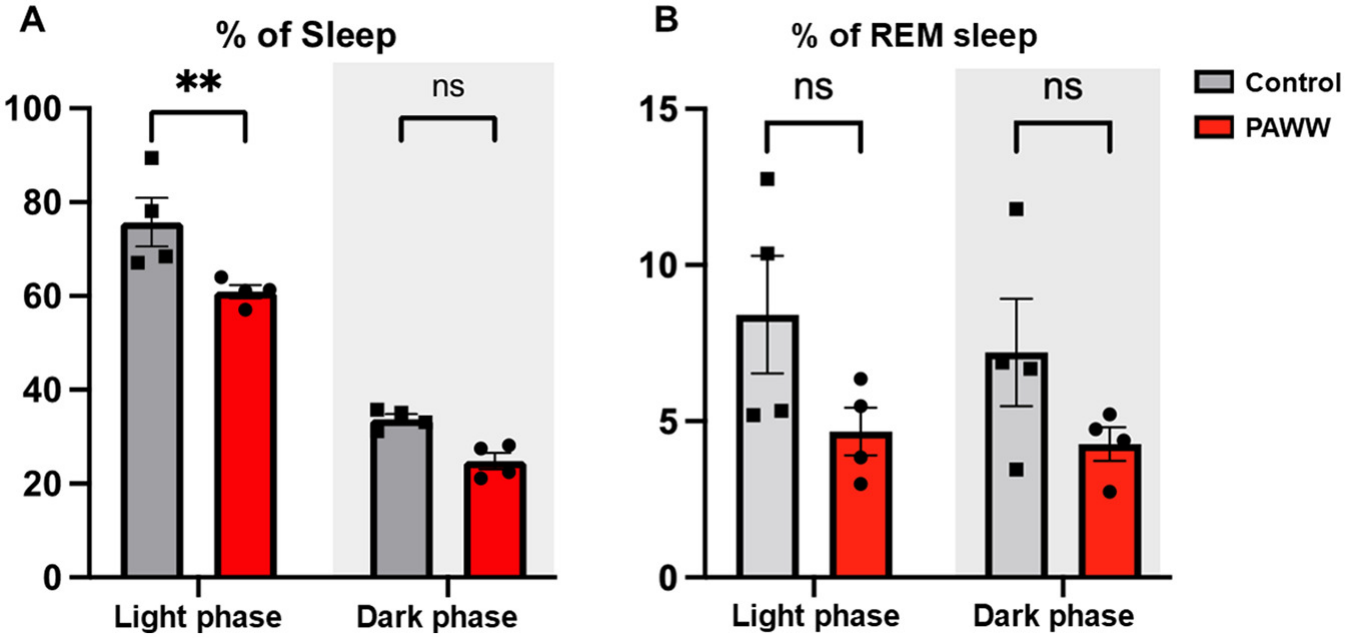
The percentage of sleep time of the mice in the PAWW and regular cages. The percentage of sleep time **(A)** and the percentage of REM sleep during sleep time **(B)** were calculated from EEG/EMG waves during the light phase (7:00–19:00) and dark phase (19:00–7:00) after 1 week of habituation and 2 weeks of housing in either PAWW cages (*n* = 4) or regular cages (*n* = 4). The results are presented as bar graphs. All data are expressed as the mean ± standard error of the mean. Repeated measures two-way ANOVA was used for statistical tests, and Bonferroni's multiple comparison test was performed as a post hoc test. The results were considered statistically significant at **P < 0.01. PAWW, perpetual avoidance of water on a wheel.

In experiment 2 (Figure 1B), in the sleep percentage evaluation by EEG/EMG, a repeated-measures two-way ANOVA revealed a significant effect of the within-group comparisons [F(1, 6) = 237.81, *P* < 0.0001, *η*² = 0.86], and the effect of the between-group comparisons was also significant [F(1, 6) = 14.11, *P* < 0.01, *η*² = 0.080], but the interaction between phase and group was not significant [F(1, 6) = 1.36, *P* = 0.29, *η*² = 0.0049]. *post hoc* Bonferroni tests revealed that the PAWW cage group (*n* = 4) exhibited a reduction in total sleep time (60.9 ± 1.4%) during the light phase, compared to the regular cage group (*n* = 4, 75.8 ± 5.2%; *P* < 0.01) (Figure 3A). There was no significant difference between the PAWW cage group (24.8 ± 1.7%) and the regular cage group (33.8 ± 1.0%) in total sleep time during the dark phase.

In the evaluation of the REM sleep percentage, a repeated-measures two-way ANOVA revealed that there were no significant effects of either phase [*F*(1, 6) = 2.00, *P* = 0.21, *η*² = 0.019] or group [*F*(1, 6) = 3.31, *P* = 0.12, *η*² = 0.33]. The interaction between phase and group was also not significant [*F*(1, 6) = 0.52, *P* = 0.50, *η*² = 0.0049]. *post hoc* Bonferroni tests revealed that the PAWW cage group also showed a lower percentage of REM sleep during the light phase (4.7 ± 0.8%) compared to the regular cage group (8.9 ± 2.1%), although this difference was not statistically significant (Figure 3B). During the dark phase, the percentage of REM sleep was 4.3 ± 0.5% in the PAWW cage group and 7.7 ± 1.9% in the regular cage group, with no significant difference between them.

**Figure 4:**
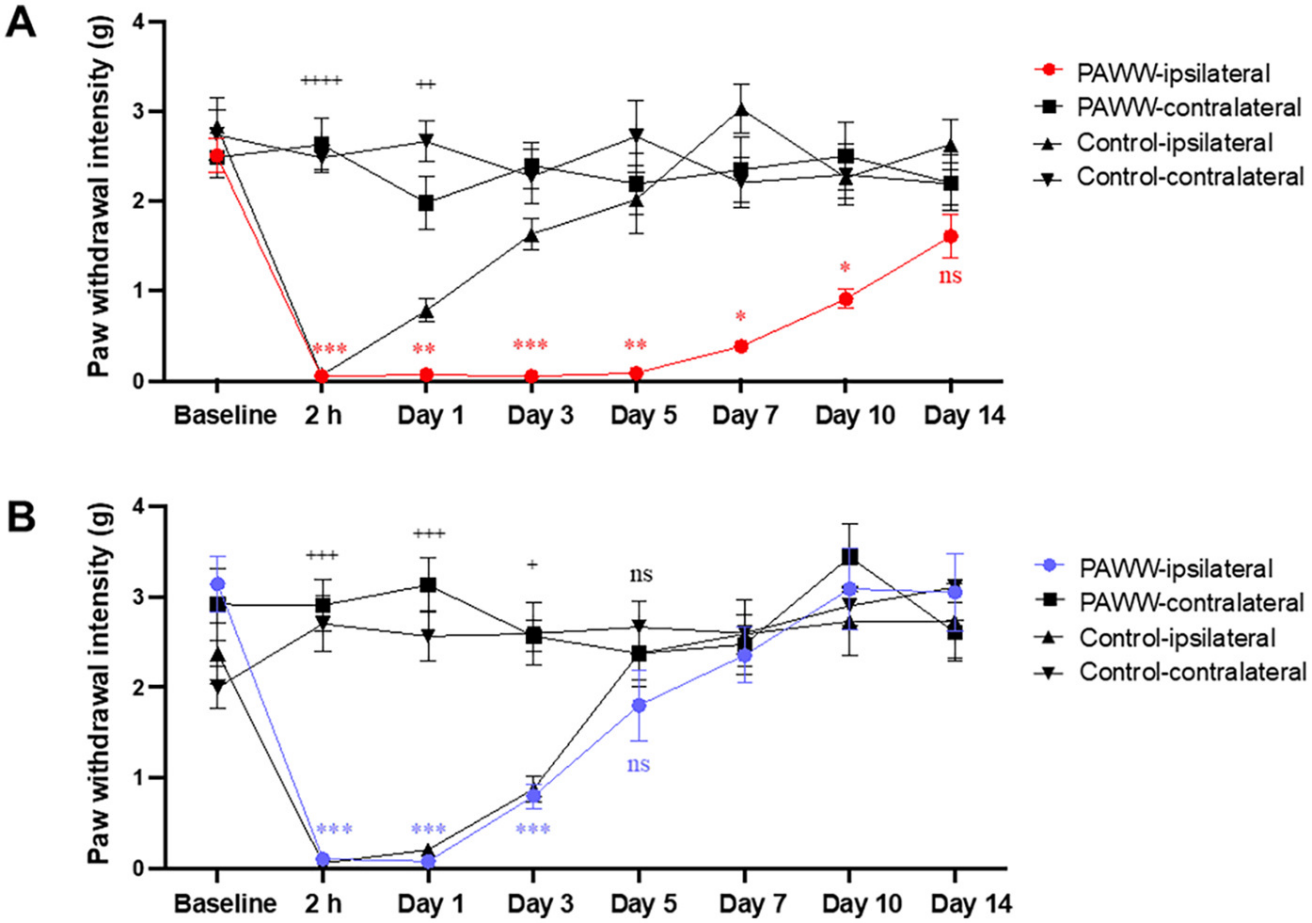
Effects of nor-BNI on the time course of mechanical pain threshold in the perioperative period. Paw withdrawal intensity was calculated using the up–down method of von Frey tests at specific test points before and after plantar incision surgery in mice housed in either PAWW cages (*n* = 8) or regular cages (*n* = 8) **(A)** Paw withdrawal intensity was also evaluated following nor-BNI treatment, administered as required, to mice in either PAWW cages (*n* = 8) or regular cages (*n* = 8) **(B)** All data are expressed as the mean ± standard error of the mean. *P < 0.05, **P < 0.01, and ***P < 0.001 for comparisons between PAWW-ipsilateral and PAWW-contralateral, and ^+^*P* < 0.05, ^++^*P* < 0.01, ^+++^*P* < 0.001, and ^++++^*P* < 0.0001 for comparisons between control-ipsilateral and control-contralateral, as assessed by repeated measures two-way ANOVA with Bonferroni's multiple comparison test as a *post hoc* test. nor-BNI, nor-binaltorphimine; PAWW, perpetual avoidance of water on a wheel.

In Experiment 3 (Figure 1C), following the plantar incision, pain behavior was assessed using the von Frey test. A repeated-measures two-way ANOVA revealed there were significant effects of groups [F(3, 28) = 55.64, p < 0.0001, *η*² = 0.35], and days [F(7, 196) = 12.90, *p* < 0.0001, *η*² = 0.15]. A significant interaction effect between days and groups was also observed [F(21, 196) = 6.80, *p* < 0.0001, *η*² = 0.20]. *post hoc* Bonferroni tests revealed that in the regular cage group (*n* = 8), the pain threshold in the ipsilateral hind paw decreased until post-surgery day 1, followed by recovery starting on day 3. In contrast, the PAWW cage group (*n* = 8) exhibited a prolonged decrease in pain threshold, with no statistically significant difference between the ipsilateral and contralateral hind paws two weeks after surgery (Figure 4A, Supplementary Table S1A).

In Experiment 4, nor-BNI was administered at three time points, followed by von Frey tests to evaluate the course of postoperative pain recovery (Figure 1D). A repeated-measures two-way ANOVA revealed there were significant effects of groups [F(3, 28) = 17.22, *p* < 0.0001, *η*² = 0.14], and days [F(7, 196) = 18.41, *p* < 0.0001, *η*² = 0.21]. A significant interaction effect between days and groups was also observed [F(21, 196) = 6.95, *p* < 0.0001, *η*² = 0.24]. *post hoc* Bonferroni tests revealed that in the regular cage group (*n* = 8), the pain threshold in the ipsilateral hind paw decreased until post-surgery day 3, followed by recovery starting on day 5. Similarly, the PAWW cage group (*n* = 8) exhibited a decrease in pain threshold up to day 3, with recovery beginning on day 5 (Figure 4B, Supplementary Table S1B).

## 4 Discussion

Our findings demonstrate that housing mice in a PAWW cage induces sleep disturbances and delays recovery from postoperative pain following a plantar incision. Notably, nor-BNI, a KOR antagonist, alleviated the delayed recovery of the mechanical pain threshold.

In our study, PAWW housing significantly increased spontaneous activity during the light phase and decreased activity during the dark phase, disrupting circadian locomotor rhythms consistent with previous reports (7). EEG recordings confirmed a significant reduction in total sleep time during the light phase, with a decreasing trend also observed during the dark phase. The mismatch observed during the dark phase, where physical activity is reduced but sleep also tends to decrease, is not merely a sign of hyperactivity, but rather reflects a broader dysregulation of sleep homeostasis. Similar patterns have been reported in human studies, in which chronic stress leads to reductions in both sleep and physical activity (31).

Compared to traditional sleep deprivation models, PAWW offers a more physiological approach to mimicking chronic sleep disturbance (Table 1). The single platform-on-water model and bar rotation method effectively induce sleep deprivation, but cause excessive acute stress and severe physical impairment, limiting their feasibility in long-term studies (4–6, 32–34). The social defeat model induces fragmented sleep and decreased pain thresholds, but has low reproducibility for sleep disturbances and primarily relies on short-term acute stress exposure (35, 36). The PAWW model, in contrast, induces chronic sleep-wake cycle disruptions without excessive acute stress or severe physical distress. It effectively mimics the circadian rhythm dysregulation seen in human sleep disorders, with characteristic alterations in corticosterone secretion rhythms and weight loss despite increased appetite (7).

**Table 1:** Comparison of sleep, pain, and other stress responses in various animal models of sleep disturbance.

Parameter	PAWW	Single platform-on-water	Bar rotations	Social defeat stress
Sleep	Mildly disrupted	REM-dominant deprivation	Total deprivation	Fragmented
Pain	Delayed recovery	Delayed recovery	Delayed recovery	Decreased pain threshold
Stress exposure period	>3 weeks	3 days	2 days	1 h/day, several times
Corticosterone	Slightly increased	Increased	Increased	Increased
Activity	Decreased	Restricted	Decreased	Decreased
Food intake	Increased	Increased	NA	Increased
Body weight	Decreased	Decreased	NA	Decreased
References	7	4, 15	6, 16	18, 19

NA, not available.

Poor sleep quality is a well-established risk factor for hyperalgesia and spontaneous pain (37–39). Several mechanisms may underlie this relationship, including opioid system dysfunction (12, 13), altered orexin signaling (18, 19), monoaminergic imbalance (serotonin, noradrenaline, dopamine) (16, 17), and immune and endocrine systems (40). Sleep disruption might delay tissue recovery, since sleep-wake cycles are essential regulators of immune function and tissue repair (41). In our study, PAWW-induced sleep disturbances prolonged postoperative pain recovery, further supporting this link. However, since sleep and stress are interdependent, their relative contributions remain uncertain. While measuring corticosterone levels could have provided insights into stress involvement, we opted not to include this measurement in this study, in order to minimize additional handling stress that could have confounded the results. Future research should include stress biomarkers (e.g., corticosterone, pro-inflammatory cytokines) to clarify the individual contributions of sleep disruption and stress-induced hyperalgesia. Exploring dual-targeted interventions may offer new therapeutic insights.

These findings may have clinical relevance for perioperative patients, who are highly susceptible to stress and sleep disturbances. Addressing sleep disturbances could be key to improving postoperative recovery. However, currently, only a few well-evidenced interventions have addressed sleep disorders in this context (42). Mice and humans share a 24 h circadian rhythm, and their sleep influences the nervous and endocrine systems similarly in both species (43). However, key differences exist: mice are nocturnal, have shorter sleep cycles (∼10–15 min between NREM and REM sleep), and exhibit polyphasic sleep patterns. These differences highlight the need for further clinical validation when translating findings from rodent models to human patients. Nonetheless, our results strongly suggest that targeting sleep disturbances might be a promising approach to improving postoperative pain recovery.

Nor-BNI is unlikely to have direct and immediate analgesic effects, since no improvement in pain thresholds was observed between 2 and 24 h post-administration. Previous studies also report a delayed onset of nor-BNI's effects (24, 44), likely due to indirect modulation of stress, sleep architecture, and pain processing (24, 25, 44, 45). Nor-BNI may also enhance wound healing by improving sleep and immune responses, both of which are closely linked to pain recovery processes (41). Although nor-BNI improved postoperative pain recovery, its underlying mechanisms remain uncertain. Given prior evidence that KOR antagonists normalize sleep in chronic pain models (8), further EEG analyses post-nor-BNI are needed to clarify whether sleep restoration mediates its effects. While we focused on KOR antagonism, it remains possible that nor-BNI's effects on pain recovery involve interactions with other neurotransmitter systems, including serotonin and dopamine (46, 47), both of which are essential for pain modulation. Tricyclic antidepressants, which enhance monoaminergic transmission, are widely used for chronic pain treatment. Thus, nor-BNI's effects may involve downstream interactions with monoaminergic systems. Future studies should quantify changes in these neurotransmitters following nor-BNI treatment and assess the therapeutic potential of combining KOR antagonists with monoamine modulators.

In addition to central mechanisms, peripheral KOR signaling may also modulate pain. KORs in peripheral sensory neurons have been implicated in nociceptive processing (48). While peripheral KOR agonists suppress inflammation and hypersensitivity, they may also contribute to opioid tolerance and hyperalgesia. Our study did not evaluate local KOR expression or inflammatory markers. Further investigation could elucidate the peripheral contributions of KOR antagonism. Because nor-BNI was administered intraperitoneally, it may have affected both central and peripheral KORs. Clarifying the site-specific contributions will require future studies employing localized drug delivery (e.g., intracerebral injection) or region-specific genetic approaches such as conditional knockouts.

While testing a KOR agonist could have provided further insights, our primary aim was to investigate the efficacy of KOR blockade. Nor-BNI was selected due to its long-lasting action, ensuring sustained receptor inhibition. Previous literature has reported that KOR activation can induce mechanical hypersensitivity (24, 25), and KOR agonists can disrupt sleep (10), suggesting that KOR agonists may exacerbate pain recovery. These findings support the rationale for targeting KOR antagonism in our experimental context. We acknowledge this as an important future direction, and we need to explore the differential effects of KOR agonists vs. antagonists on sleep-related pain outcomes in subsequent studies. Further investigations are required to determine whether the observed effects are specific to KOR blockade or involve additional neurotransmitter systems. Future studies incorporating KOR agonists, monoamine modulators, and glutamate antagonists may clarify the complex mechanisms underlying sleep-related pain enhancement.

**T**he limitations of this study include the short duration of observation for postoperative pain recovery and sleep disturbances. While we demonstrated that sleep disruption delays pain recovery, we could not assess long-term consequences, such as chronic pain development, due to the limited observation period. Future studies with extended monitoring periods are needed to evaluate potential long-term effects. Additionally, although we observed a trend toward REM sleep reduction in the PAWW group, this difference did not reach statistical significance. This suggests that a larger sample size or more precise EEG analysis methods might be necessary for better assessment of REM sleep alterations and their potential impact on pain recovery. Furthermore, while nor-BNI improved postoperative pain recovery, the underlying mechanisms remain unclear. Future studies should include EEG assessments following nor-BNI treatment, along with investigations into other potential mechanisms, such as monoaminergic or neuroimmune interactions, to elucidate the precise role of KOR antagonism in postoperative pain recovery. Additionally, our study utilized only male mice to minimize variability related to the estrous cycle and to maintain consistency with prior PAWW model studies. Future research is warranted to determine whether similar effects are observed in female mice, which may provide further insight into sex-specific mechanisms in sleep–pain interactions and KOR signaling.

In conclusion, our study demonstrates that the PAWW model, which mimics sleep disorders in modern society, delays recovery from postoperative pain. Importantly, our findings suggest that KOR antagonism could be a promising option to prevent or treat prolonged pain induced by sleep disorders in perioperative patients. This potential application of KOR antagonists in perioperative care is the key takeaway of our research.

## Data Availability

The raw data supporting the conclusions of this article will be made available by the authors, without undue reservation.
