# Supplementary figures and images for: Effects of a kappa opioid receptor antagonist on delayed postoperative pain recovery in a novel mouse sleep disorder model
Source: Front Pain Res (Lausanne). 2025 Jul 4;6:1516935. doi: 10.3389/fpain.2025.1516935 (PMC12271097; doi:10.3389/fpain.2025.1516935)

**Supplementary Table 1. Mechanical pain thresholds in Experiments 3 and 4**

**
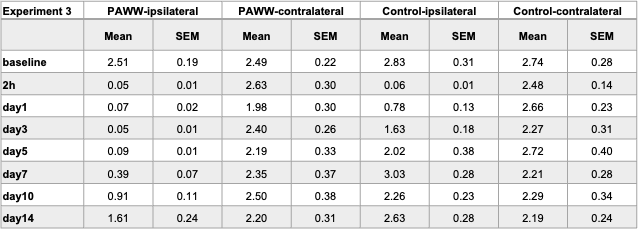
a**

**
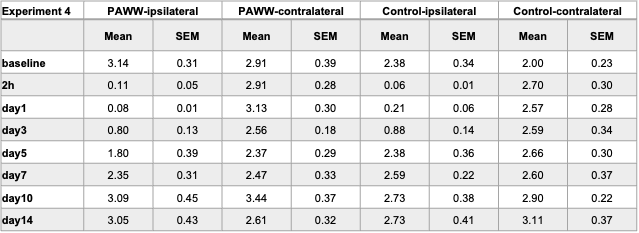
b**

Supplement: Supplementary file 1 [file Datasheet1.docx]
